# Aerobic Fitness and Cognitive Functions in Economically Underprivileged Children Aged 7-9 Years: A preliminary Study from South India

**Published:** 2011-03

**Authors:** Arpitha Jacob, Crystal D. D’Souza, S. Sumithra, Sandhya Avadhani, Chaya Mayasandra Subramanya, Krishnamachari Srinivasan

**Affiliations:** 1 *St. Johns Research Institute, Bangalore-560034, India;*; 2 *St. Johns Medical College and Hospital, Bangalore-560034, India;*; 3 *Swami Vivekananda Yoga Anusandhana Samsthana, Bangalore-560019, India*

**Keywords:** aerobic fitness, cognitive functions, economically underprivileged children, South India

## Abstract

This study examined the relationship between aerobic fitness and cognitive functions in 7-9 year old school going children hailing from a socio-economically disadvantaged background in Bangalore, India. Ninety eight children (51% boys and 49% girls) were assessed on height, weight, BMI, aerobic fitness (multistage 20 m shuttle test) and cognitive functions (verbal tests: comprehension, arithmetic, vocabulary, analogies; performance tests: block design, object assembly and coding). Number of shuttles was significantly positively correlated with two of the cognitive tests: comprehension (*p*=0.01) and block design (*p*=0.005). Multiple linear regression analysis showed that the number of shuttles emerged as an independent predictor of tests of comprehension and block design after adjusting for BMI and gender. The above findings provide preliminary evidence for the association between aerobic fitness and cognitive functions in children from poor socio-economic background.

## INTRODUCTION

A recent meta-analysis on school based physical activity and cognition showed that physical activity had a positive influence on concentration, memory and classroom behavior (1). Experimental studies using either a cross sectional design or test-post test comparison demonstrated a significant positive relationship between physical activity and cognitive performance in children (2). A recent review examining the effects of aerobic exercise on various cognitive tasks found that the optimal intensity for impacting cognitive tasks covered a wide range (~40–80% VO_2_ max) and exercise duration of more than 20 minutes was most efficient in increasing the performance on perceptual and decisional tasks (3). However, a meta-regression analysis concluded that the empirical literature did not support a link between cardiovascular physical fitness and cognitive performance (4), but the number of studies that had young children as subjects was very small. In the last two decades there has been a resurgence of interest in the area of physical fitness, cognitive and academic performance in children and adolescents. In a recent randomized controlled trial of aerobic exercise training on cognition in overweight children, there was a significant improvement in executive functions in children in the high dose exercise group compared to controls (5). Most of the published studies on physical exercise and cognition in children have come from the West with few published studies from developing countries. To the best of our knowledge there are no known published Indian studies examining physical activity and cognition in school children from an economically disadvantaged background. In the present study, we examined the association between aerobic fitness and cognitive functions in 7-9 year old school going children from a socio-economically disadvantaged background living in Bangalore, India.

## METHODS

The sample size consisted of hundred, 7-9 year old healthy (as assessed by clinical examination by medical professionals) school going children hailing from a socio-economically disadvantaged background (average monthly income of 2000 INR, equivalent to 46 USD approximately). All participants were recruited from a single school in Urban Bangalore, India. From a total of 200 children who were part of a larger interventional study on the effects of yoga practices on cognitive performance, physical fitness was assessed in 100 randomly selected children at baseline before the start of yoga intervention. The children gave oral assent while the parents/legal guardian provided written informed consent. The school also provided written permission to conduct the study on its children, on the school premises. The study was approved by the Institutional Ethical Review Board of St. John’s National Academy of Health Sciences. Socio-demographic details were obtained from all children. Height, weight and BMI (Body Mass Index) were recorded. Children underwent a physical examination and apparently healthy children with no history of chronic diseases, physical or mental handicap and not severely undernourished (<-3SD for weight for age and -3SD height for age z scores of the National center for health statistics / WHO standards) (6) were invited to participate in the study. The Indian adaptation of WISC II, Malin’s Intelligence Scale for Indian Children (7) was used to measure cognitive performance. The test contains both verbal and performance subtests. For the purpose of the present study, 4 verbal, and 3 performance tests from the battery of tests were used. The verbal tests were comprehension, arithmetic, vocabulary and analogies. Block design, object assembly and coding were the performance tasks. The tests were administered by trained psychologists in the morning hours.

The multistage 20 m shuttle test described by Leger and Lambert (8) was used as an index of physical fitness. The children were required to run continuously between two points which were 20 m apart. The pace of running was indicated by an audio recording which emitted beeps at prescribed intervals. The initial speed was set at 4 km/h and increased by 0.5 km/h for each subsequent minute. The test was discontinued when the child voluntarily stopped due to fatigue. The total number of laps completed was used as an index of physical fitness. Previous research has shown that the number of laps completed positively correlates with VO_2_ max (9). All study assessments were conducted in the school premises. At the time of conducting test of aerobic fitness all children, as part of the school curriculum, were doing physical exercises such as running and stretching exercises for about 30 minutes twice a week.

### Statistical Analysis

Analyses were done using the SPSS (version 17) software. Continuous variables were reported using mean (SD) and the categorical variables were reported using frequencies and percentages. Non normal data was log transformed. Pearson correlation coefficient was computed to assess the association between the cognitive measures and the number of shuttles. Multiple linear regression analysis was computed to identify the predictors of cognitive measures. In the regression analysis BMI and sex were adjusted for since these factors have been reported to influence aerobic functioning. All analysis was considered statistically significant at the 0.05 level of significance.

## RESULTS

Of the 100 children enrolled in the study, 98 children completed both the assessments (physical fitness and cognitive tests); both genders were almost equally distributed (boys=51%). The children hailed from the lower socio-economic strata with average parental income of Rs, 2000 per month (US $46). Most of the parents were employed as daily wage laborers and were illiterate. The mean age of the sample was 7.9 ± 0.9 yrs. The mean height, weight and BMI of the sample were 1.21 ± 0.07 m, 20.4 ± 3.06 Kg and 13.8 ± 1.1 respectively.

For the analysis the number of shuttles was used as the indicator of physical fitness. The average number of shuttles completed was 46.1 ± 14.2. In the verbal tests of comprehension, arithmetic, analogies and vocabulary the mean scores were 7 ± 2.4, 5.2 ± 1.9, 6.2 ± 3.2 and 13.1 ± 4.4 respectively. The mean scores for the various performance tests that included block design, object assembly and coding were as follows: 6.2 ± 4.1, 4.5 ± 2.5, and 32.4 ± 8.1 (Table 1).

**Table 1:** Descriptive data of sample characteristics, number of shuttles and cognitive variables

Variables	Mean	SD

Age (years)	7.9	0.9
Height (metres)	1.21	0.07
Weight (Kg)	20.4	3.06
BMI	13.8	1.1
Number of Shuttles	46.1	14.2
Comprehension	7	2.4
Arithmetic	5.2	1.9
Analogies	6.2	3.2
Vocabulary	13.1	4.4
Block Design	6.2	4.1
Object Assembly	4.5	2.5
Coding	32.4	8.1

The number of shuttles was significantly positively correlated with two of the cognitive tests: comprehension (*P*=0.01) and block design (*P*=0.005) (Table 2). Multiple linear regression analysis showed that the number of shuttles is an independent predictor of tests of comprehension and block design after adjusting for BMI and gender. Results indicated that number of shuttles explained 8% of the variance in comprehension and block design scores respectively (Table 3).

**Table 2:** Correlation between number of shuttles and cognitive variables

Variable	Correlation	P value

Comprehension	0.249	0.01
Arithmetic	0.187	0.06
Analogies	0.138	0.17
Vocabulary	0.140	0.17
Block Design	0.283	0.005
Object Assembly	0.126	0.215
Coding	-0.138	0.176
BMI^a^	0.11	0.28

a Body Mass Index.

**Table 3:** Results of the multivariate linear regression analysis

Variable	B coefficient	Adj R^2^	*P* value	95% C. I.	
				LL	UL

Comprehension	0.039	0.08	0.025	0.005	0.074
Block design	0.009	0.09	0.008	0.002	0.016

## DISCUSSION

The main objective of the study was to explore the association between aerobic fitness and cognitive functions in 7-9 year old children from a poor socio-economic background. The study findings indicated a positive association between aerobic capacity as measured by shuttle tests and cognitive functions in 7-9 year old children. This is corroborated by previous research where children who are physically fit perform better and faster on cognitive tests than children who are less fit (10, 11) and our study extends this finding to school going children from a disadvantaged background.

Among the various cognitive tests, significant positive association was found between aerobic capacity and cognitive measures of both verbal (comprehension) and performance tasks (block design). Previous studies have shown that particular types of cognitive abilities are sensitive to benefits of aerobic fitness (2, 12). Though the exact mechanistic pathways through which physical fitness impacts cognitive functions have not been ascertained, various explanations have been put forward. A child’s fitness may reflect the child’s overall health, which in turn may positively impact the child’s cognitive performance (13). In addition, movement particularly in young children stimulates cognitive development (14). Recent research has also shed light on the possible neural mechanisms involved. Animal studies have shown that aerobic activity increased capillary blood flow to the cortex and promote growth of new neurons and synapses, resulting in better performance (15, 16).

The findings in this small study of a modest positive association between physical fitness and cognitive measures among school going children from economically disadvantaged background is in agreement with earlier studies. Future studies on larger sample of children with more comprehensive measures of both physical fitness and cognitive functions are clearly needed including exploring dose-effect relationship between physical fitness and cognitive performance. This is especially important given that a large number of children from developing countries fail to reach their optimal cognitive potential (17) and introduction of regular physical activity in schools may be a cost effective method of overcoming this significant problem.
